# “Media, politics and science policy: MS and evidence from the CCSVI Trenches”

**DOI:** 10.1186/1472-6939-14-6

**Published:** 2013-02-12

**Authors:** Daryl Pullman, Amy Zarzeczny, André Picard

**Affiliations:** 1 Division of Community Health and Humanities, Faculty of Medicine, Memorial University, 300 Prince Philip Drive, St. John’s, NL, A1B 3V6, Canada; 2 University of Regina, 110, 2 Research Drive, Regina, SK, S4S 0A2, Canada; 3 The Globe and Mail, 444 Front St. W, Toronto, ON, M5V 2S9, Canada

**Keywords:** Multiple sclerosis, CCSVI, Liberation therapy, Priority setting, Public pressure, Media, Politics, Evidence

## Abstract

**Background:**

In 2009, Dr. Paolo Zamboni proposed chronic cerebrospinal venous insufficiency (CCSVI) as a possible cause of multiple sclerosis (MS). Although his theory and the associated treatment (“liberation therapy”) received little more than passing interest in the international scientific and medical communities, his ideas became the source of tremendous public and political tension in Canada. The story moved rapidly from mainstream media to social networking sites. CCSVI and liberation therapy swiftly garnered support among patients and triggered remarkable and relentless advocacy efforts. Policy makers have responded in a variety of ways to the public’s call for action.

**Discussion:**

We present three different perspectives on this evolving story, that of a health journalist who played a key role in the media coverage of this issue, that of a health law and policy scholar who has closely observed the unfolding public policy developments across the country, and that of a medical ethicist who sits on an expert panel convened by the MS Society of Canada and the Canadian Institutes of Health Research to assess the evidence as it emerges.

**Summary:**

This story raises important questions about resource allocation and priority setting in scientific research and science policy. The growing power of social media represents a new level of citizen engagement and advocacy, and emphasizes the importance of open debate about the basis on which such policy choices are made. It also highlights the different ways evidence may be understood, valued and utilized by various stakeholders and further emphasizes calls to improve science communication so as to support balanced and informed decision-making.

## Background

Science policy in general and health policy in particular emphasize the necessity of being evidence based. At the same time, the need to engage members of the public in open discussion about fundamental issues that could affect their well-being has been gaining traction in recent years. “Public participation,” “citizen engagement,” “deliberative democracy,” and related notions are now part of the science and health policy lexicons. But it is one thing to engage citizens about a particular policy issue, such as whether they would participate in a biobank, and if so, what in their view would constitute appropriate consent [1,2]. It is quite another to have the public at large set the policy agenda by first dictating specific questions that science should address, and then passing judgment upon what constitutes “evidence” in support of a particular conclusion. The advent of social media in recent years is changing the dynamics of the relationship between scientists, politicians, health professionals and the public at large. Special interest groups now use these tools to gather and disseminate information about key issues, and to exert pressure on various bodies to advance their agendas. The broader implications this growing trend has for what constitutes evidence based science and health policy are just beginning to emerge.

This paper addresses some aspects of this dynamic as it has played out over the past three years in Canada with regard to a controversial new treatment for multiple sclerosis (MS). MS is a neurodegenerative disease involving inflammation and degeneration of myelin, the protective covering around the nerve cells in the brain and spinal cord. There is no consensus about its cause. While MS is often characterized as an autoimmune disorder, other potential theories relate to toxins, environmental triggers, vitamin D deficiency, infectious agents, genetics and vascular abnormalities. Common symptoms include visual disturbance, speech problems, numbness, pain, loss of balance, loss of coordination, bladder and bowel problems, stiffness, weakness, paralysis and fatigue. Symptoms vary widely from person to person and throughout the course of the disease. While current treatments can decrease the severity and frequency of MS attacks and may slow disease progression, many treatments are associated with significant side-effects. There is no proven cure [3].

Given the potentially devastating impact of MS and the continuing bewilderment and speculation as to its cause, it is little wonder that general excitement ensued when Dr. Paolo Zamboni, an Italian vascular surgeon, announced he may have discovered a key cause of the disease. Based on the results of a study conducted in his clinic, Zamboni theorized that MS is caused by narrowed or blocked veins in the neck which prevent the efficient removal of blood from the brain and spinal cord. Zamboni speculates this condition leads to a build-up of iron, which in turn triggers the inflammation and myelin degeneration seen in MS sufferers [4]. Chronic cerebrospinal venous insufficiency (CCSVI) is now part of the MS vernacular to describe this putative cause. CCSVI is diagnosed by ultrasound and treated surgically by balloon angioplasty, or by the insertion of stents to keep the veins open. Zamboni coined the term “liberation therapy” to describe this unprecedented treatment, and has called for randomized trials to assess its effects more rigorously.

Zamboni’s theory was contentious from the beginning, in part because the initial study that received so much attention was a non-randomized, non-blinded study of 65 patients. With no controls for placebo effect or to account for spontaneous improvements common to the relapsing-remitting form of MS, there was much skepticism in scientific and medical communities as to the validity of his results. Indeed subsequent studies were unable to replicate Zamboni’s original findings [5,6]. Concerns were raised as well about potential risks associated with the proposed therapy including possible hemorrhage, dislodgment of blood clots resulting in heart attack or stroke, nerve damage in the neck, complications at the puncture site and vessel puncture by the catheter, among others [7]. Nevertheless, clinics around the world started offering liberation therapy [8], and many desperate MS sufferers became medical tourists, investing considerable time, effort and financial resources in the hope of a cure [9].

In most jurisdictions, mainstream medicine has treated Zamboni’s proposed diagnosis and treatment as little more than a curiosity. Such has not been the case in Canada. For reasons that are still not entirely clear, CCSVI has become a rallying point for many in the Canadian MS community. Despite the risks and the equivocal state of the evidence, the suggestion that MS may have physiological origins with a surgical solution has triggered a flurry of media coverage [10], ignited the hopes of patients and their families, and divided the community of MS specialists [11]. The MS Society of Canada, the Canadian Medical Association, and the Canadian Institutes of Health Research (CIHR) have all urged caution and the need for further investigations to establish a solid evidentiary basis for the CCSVI diagnosis and liberation therapy. Nevertheless, countless MS patients and their supporters in Canada have formed advocacy groups and used the internet and social media to advocate for clinical trials and in support of venoplasty as a treatment they insist should be covered under the publicly funded health care system [8]. Advocates often attack the credibility of those who express skepticism about CCSVI, or who call for a cautionary approach [10]. It has been suggested that “[p]erhaps for no other condition has social media been as effective in promoting a medical theory as with chronic cerebrospinal venous insufficiency” [12].

In what follows we share some of our experiences and insights with regard to the CCSVI/Liberation Therapy phenomenon as it continues to unfold in Canada. We characterize our perspectives as “from the trenches” as each of us, to some degree, has played a role or otherwise been engaged in this continuing saga as it has unfolded in the Canadian context. AP is a health journalist for the *Globe and Mail*, Canada’s national newspaper. He played a key role in this breaking story and has commented extensively on it as it has continued to unfold. AZ is a health law and policy expert located in Saskatchewan, the Canadian province that has acted most aggressively with regard to CCSVI. DP is a member of an expert panel convened by the MS Society of Canada and the CIHR to oversee a number of studies designed ostensibly to assess the CCSVI-MS hypothesis and to establish whether an evidentiary basis exists to justify proceeding with a clinical trial. The work of this panel has been closely scrutinized and the political pressure to proceed with a clinical trial has been palpable.

## Discussion

### Politics and publicity—André Picard

On November 21, 2009, the CTV network’s flagship newsmagazine broadcast a story entitled “The Liberation Treatment: A Whole New Approach to MS” [13]. The same day, The *Globe and Mail* (part of the same media conglomerate) published a companion story entitled “Researcher’s labour of love leads to MS breakthrough” based on the documentary [14]. It was not your typical 90 second TV news story, but a 30-minute documentary, months in the making. The story focused on Zamboni’s work and the timing of the documentary was pegged to the publication of his research in a peer-reviewed journal, but also emphasized the source of the doctor’s quest to find a cure—his wife is afflicted with MS. The CTV documentary had all the elements of a good news, labor of love story.

Zamboni became interested in MS in 1999 when his wife was diagnosed with the condition. He set out to learn all he could about MS and was initially intrigued about repeated references, dating back a century, to the possibility that iron build-up might be a contributing factor. Zamboni’s primary area of research was related to how heavy metals like iron damage blood vessels in the leg; he wondered if there could be a similar problem with blood vessels in the brain. Using ultrasound scans, Zamboni discovered vein blockages and abnormalities in all the MS patients he tested. He wondered if clearing blockages in the neck could alleviate symptoms and received ethics approval to conduct a procedure similar to angioplasty on patients. The CTV documentary showed patients before, during, and after the surgery, their MS symptoms seemingly alleviated. Even Zamboni’s wife had the surgery, giving the story a lovely romantic touch.

So, yes, the initial story about CCSVI was powerful and enthusiastic. But the story also cautioned that “evidence is too scant and speculative to start rewriting medical textbooks” and that “MS sufferers should not rush off to get the surgery” [14]. Missing, however, were images of skeptics and opponents of the CCSVI theory. The MS Society of Canada refused to go on camera, instead issuing a short written statement urging caution. Some physicians who specialize in MS believed the theory was quackery but did not want to be quoted for fear of giving the idea any credence. They would later become the fiercest critics of the media treatment of the story.

Where CCSVI really took off though was in the blogosphere. There, the provisos evaporated and the “liberation” treatment was billed as a miracle cure. Testimonials continued to pile up.

Patients began to clamor for the surgery and understandably so, as traditional treatments for MS are often frustratingly ineffective. Many were willing to invest their life savings, and clinics—in Poland, in Costa Rica, in Bulgaria—were more than happy to take their money. The initial reports from patients fuelled the hype, and increasingly breathless media coverage. Those crippled by MS began walking, seemingly on water. Those who urged caution were shouted down, often dismissed as pawns for Big Pharma. There were demands for the liberation procedure to be funded in Canada. Health ministers vowed to finance research.

Meanwhile, science took its course, and so did journalism. Researchers cautiously tried to reproduce Dr. Zamboni’s initial findings. Zamboni himself joined the chorus urging a go-slow approach; while he was enthusiastic about the procedure he cautioned that vein blockage was not the sole cause of MS and urged more research to better understand the potential risks and benefits. These developments were chronicled in some detail in the mainstream press [15].

Then less rosy reports about liberation surgery began to surface. Veins collapsed in about half of those who underwent angioplasty and stents were required. Patients initially felt better. Then, weeks after the procedure, blockages reappeared; many suffered dangerous blood clots, a common side effect of stenting. In November 2010, Mahir Mostic, a 35-year-old Canadian, died after undergoing the procedure [16]. That was a turning point. Subsequent media coverage became both more cautious and more skeptical.

But CCSVI had momentum, particularly political momentum. There has been much criticism of elected officials for funding CCSVI research. But they were under tremendous pressure; MS patients are well-organized, vocal and, in many cases, desperate. Given that the traditional treatment for MS involves very expensive drugs, one of the powerful undercurrents of the story was a belief that those who opposed further research were in the back pocket of Big Pharma. Time and again we were reminded that the researchers who discovered that stomach ulcers were caused by H. pylori bacteria were treated as quacks by mainstream medicine, and especially by surgeons who made a living doing ulcer surgery.

There is also, in the CCSVI narrative, a belief that there are two camps: MS patients pushing for an unproven intervention and scientists who opposed them. The reality is more complex. Patients were deeply divided. Clinicians and scientists also had a vast array of opinions, ranging from those who believed CCSVI had some validity [17] through to those that believed research should be funded to provide a definitive answer, and on to those who held that the theory was biologically implausible. All appealed to some semblance of “evidence” to back their positions.

Of course “evidence” means starkly different things to different people. Evidence, to academics and scientific researchers, means carefully gathered data that is analyzed and interpreted through a rigorous process with established norms. This no doubt accounts in part for the cautious approach that characterized the scientific community’s initial response to this story. However, for some in the research establishment, evidence consists only of the data that supports their particular hypothesis, or serves their purposes. There are a lot of biases that taint interpretation of evidence. Let’s not forget that medical journals tend to publish only positive studies, not negative ones, particularly when it comes to research on drugs. That their advertising derives principally from pharmaceutical company ads is, no doubt, merely a happy coincidence.

For the public and patients, the most powerful evidence is often anecdotal, and the CCSVI story provided a lot of powerful anecdote, written and visual. For politicians, the most powerful evidence is too often that which is stated loudest and, again, the cries for help from the MS community were loud, and understandably so.

And what is evidence for journalists? It can be any combination of the above. Journalists love anecdote—particularly heart-wrenching stories; and they like the underdog, the driven researcher fighting the medical establishment. In this case, and with the benefit of hindsight, that approach may have led to an over-enthusiastic and inadvertent promotion of some shaky science.

Of course, having evidence is not nearly enough. You have to communicate the evidence. With CCSVI, the patients—especially those who believed in this procedure—won the communication battle, at least in the early rounds. Scientists who had their doubts did not make their arguments well, if at all. As noted previously, however, such a cautious and guarded approach is typical of the discipline. In the absence of concrete evidence to either support or debunk Zamboni’s hypothesis, scientists generally were hesitant to make any definitive pronouncements. While this hesitancy is understandable to a point, scientists and journalists alike could resolve some of these ‘cultural’ issues by making use of an interlocutor, an independent third party like the Science Media Centres that have been established in Britain, Australia, and Canada as well [18].

But, in the end, one thing that journalism and science—two disparate professions—have in common is that they are self-correcting over time. It’s not always a pretty process but it is a necessary and informative one.

### Politics and finances—Amy Zarzeczny

Canada is well known for its largely publicly funded health care system. Health is an area of shared jurisdiction between federal and provincial governments. Through the *Canada Health Act*, the federal government transfers funds to the provinces to administer their provincial health care plans. Decisions regarding availability and public coverage for health services and products occur at various levels, and although processes differ between provinces, a complex web of factors is often engaged. These decision-making processes (and their political elements) are normally distinguished from research funding decisions. As addressed in the following section, the latter are generally made by institutions and bodies with particular expertise in the area and with the mandate to manage funding programs. The Canadian MS liberation story is particularly interesting because of the degree to which it brought these different spheres together, reset the parameters, and directly engaged politicians (at the behest of the public) in the research funding realm.

Exactly why the CCSVI wave hit Canada so much harder than other countries remains an open question. Perhaps it is the fact that Canada has one of the highest rates of MS in the world, at approximately 240/100,000 people [19], or perhaps the public health care system means Canadians look more quickly to government to take responsibility for health needs. Clearly the media’s role was not insignificant, as the topic received more attention in mainstream Canadian newspapers than any other country, including even Italy [10]. By November 25, 2009, very early in this story’s evolution, an online petition entitled “Support The Liberation Procedure (The Zamboni Procedure)” which targeted Health Canada had already garnered 17,624 signatures [20]. Supporters formed province-specific Facebook groups, among other forms of social networking, to share information and advocate.

Whatever the reason for its origin and momentum, this considerable public pressure prompted action by both federal and provincial policy-makers. At the federal level Liberal Member of Parliament (MP) Kristy Duncan introduced Bill C-280, *An Act to establish a National Strategy for Chronic Cerebrospinal Venous Insufficiency* (*CCSVI*) [21]. The Bill’s preamble referenced evidence supporting CCSVI as a cause of MS and set out a framework for establishing a national strategy, including clinical trials. Although Bill C-280 was defeated on Second Reading, it brought the debate into Parliament both in a formal sense and in the political maneuvering that surrounded it [22]. The degree to which Bill C-280 reflected direct political involvement in the realm of science and medical research raises interesting questions about the priority setting process.

Perhaps partly in response to a perception that the federal government was not moving with appropriate speed in advancing CCSVI research and clinical application, several provinces acted independently to allocate funds directly [23]. British Columbia invested $700,000 over three years in a registry to track the experiences of patients who had received treatment [24]. Similarly, Alberta Health and Wellness set aside up to $1 million (all figures are reported in Canadian dollars) to support a three year observational study tracking the experiences of Albertans [25]. Interestingly, the Government of Alberta also expressed its commitment to fund clinical trials, but only “if and when it is safe and ethical to proceed” [26]. The Government of Newfoundland and Labrador provided $400,000.00 to support an observational study of 30 patients who had received liberation therapy. Using objective preliminary and post-procedural tests, the study measured changes in the mental or physical status of patients over a twelve month period, as compared to a control group of 10 who had not received the treatment. The results confirmed “there were no measurable objective medical changes in the observed patients who underwent the CCSVI procedure” [27].

Of all the provinces, Saskatchewan arguably moved the fastest and went the furthest in terms of devoting public funds for clinical research into CCSVI. Rather than taking an observational approach, Saskatchewan focused quickly on interventional studies. On October 19, 2010, the Saskatchewan Government announced a commitment of $5 million to clinical trials for liberation therapy. Its neighboring province, Manitoba, subsequently announced its intent to partner with Saskatchewan on this trial with a matching $5 million [28]. Yukon Health and Social Services similarly announced a $250,000 contribution. However, after assessing the proposed research initiative and the specific projects that were applying for funding, the Saskatchewan Health Research Foundation (SHRF)—the scientific body normally tasked with judging the nature and quality of such initiatives—concluded no proposal should be funded at that time [29]. The Saskatchewan Government was undeterred, and went on to pursue partnering opportunities in other jurisdictions. On January 12, 2012, Premier Brad Wall announced $2.2 million to allow Saskatchewan residents to participate in a clinical trial at the Albany Medical Centre in Albany, New York. Six-hundred eighty-two applications were subsequently received for an anticipated 86 spots for Saskatchewan residents [30]. The first Saskatchewan patient to participate received his surgery in August, 2012 [31].

The Saskatchewan Government’s commitment to moving clinical trials forward has not waivered, notwithstanding the lack of consensus around a number of key issues including diagnostic techniques, whether there is in fact a meaningful association between CCSVI and MS and, if so, what the nature of that association might be (e.g., cause versus effect). Indeed, in a September 23, 2011 press release, then Minister of Health Don McMorris expressed, “‘Patients need answers as soon as possible . . .,’ and, ‘. . . [w]e owe it to them to explore every opportunity to advance MS research and find answers about this treatment’” [32].

Saskatchewan’s incidence rate of MS is approximately 340/100,000 people [33] With a population of just over one million, roughly 3,400 people in Saskatchewan are living with MS. In a province often characterized by its small-town feel and highly interconnected social networks, one needn’t look far before meeting someone who is personally affected by MS, either directly, or through a loved one. Accordingly, it is perhaps no wonder this issue sparked so much public interest in the province and got the attention of its political leaders. Nevertheless it raises concerns about the relationship between science and politics and about the role of the media and social media in particular in shaping that relationship.

### Politics and science policy—Daryl Pullman

It would be naïve to believe that science proceeds independent of political priorities and pressures. Any time scientific institutions depend upon the public purse to finance their endeavors, various political priorities will influence the scientific agenda. As noted previously, however, generally such broad agenda setting occurs somewhat arms-length to the scientific process in macro-level decisions about how much of the global budget to allocate in support of scientific research. Ideally once general allocation decisions are made it is left to research institutions with the requisite knowledge, experience and expertise to make the meso and micro-level decisions for where those funds will be spent, presumably on the basis of available evidence. When politics intrudes on these more specific scientific decisions the danger is that instead of evidence guiding policy, policy and political pressure will guide what counts as evidence.

In some respects the expert panel convened in the fall of 2010 by CIHR in partnership with the MS Society of Canada, was created to help manage the emerging public and political pressures and to regain some control over the scientific agenda with regard to CCSVI. The fact that Alain Beaudet, president of the CIHR, decided to chair the panel himself, is an indication of the high priority (at least politically, if not scientifically) the file had assumed in a relatively brief period of time. In August 2010, prior to forming the expert panel, the CIHR and the MS Society convened a joint invitational meeting of top researchers “to identify priorities for Canada that would accelerate research and innovation on treatments for MS” [33]. Despite the somewhat general mandate with regard to MS treatments (plural), the focus was almost exclusively on CCSVI. Nevertheless, the summary and recommendations coming out of that meeting—including the strong recommendation to establish the expert panel—indicate a general determination to ensure that scientific rigor would guide policy decisions rather than the reverse. “Meeting participants were emphatic about the crucial requirements for strong evidence based decision making, at both medical and political levels . . .” [34]. However, what serves as evidence in medicine, may be perceived quite differently in politics. Indeed, on-going commentary on the panel’s activities indicates that one person’s “evidence” can be another’s “politics.” The subsequent activities of the expert panel and the manner in which its activities were perceived bears this out, and demonstrates just how difficult it can be to maintain a strong commitment to scientific rigor in the face of relentless public and political pressure.

Unlike other members of the expert panel, I (DP) and my colleague Bartha Knoppers—an expert in health law— are neither research scientists nor clinicians. Hence we have a somewhat unique perspective on how this process unfolded within the expert panel. While we had some sense of the political pressure on the panel in general, we did not experience the same direct pressure as did many of our clinical and scientific counter-parts. Clinician members of the panel manage MS patients, many of whom had gone abroad to receive liberation therapy and were convinced of its efficacy. Other patients were anxious to receive the therapy and were openly frustrated when they could not get it at home. Virtually all patients wanted a definitive clinical trial to prove the case once and for all. Researchers on the panel had their scientific credentials and personal integrity questioned by CCSVI advocates who often posted comments on web sites and were quoted in the media with regard to industry sponsored research in which panel members had been involved. The implication, if not explicit charge, was that panel members had a vested financial interest in dismissing CCSVI and liberation therapy as scientifically unsound so as to maintain the status quo.

In effect the expert panel functions as an oversight committee for on-going research related to venous anatomy and MS. The panel consists of the principal investigators of seven MS Society sponsored studies (four from Canada and three from the US) that were carefully designed to investigate various aspects of the CCSVI hypothesis. Other members included scientific leadership from CIHR and the MS Society, a representative from the provinces and territories, an international representative, as well as a medical ethicist and a health law expert. The mandate of the panel was to monitor and analyze preliminary and final results from the seven on-going studies and from related studies from around the world. If the accumulating data was judged to provide “clear and convincing evidence” in support of the CCSVI hypothesis, the panel could recommend that the federal government proceed with a clinical trial.

From the outset the panel’s work was closely scrutinized and critiqued. Kirsty Duncan, the MP who had called for a national CCSVI strategy complained “. . . CIHR put a political process in place instead of a scientific process. Evidence was being willfully ignored from the literature, from scientific conferences, and from returning Canadians treated for CCSVI” [22]. There is a certain irony when a politician accuses scientists of playing politics, and a degree of naiveté evident when literature, reports from scientific conferences, and anecdotal reports from MS patients are apparently placed on the same evidentiary plane. Indeed it is just such naïve views about the nature of evidence that the expert panel was designed to counter.

Nevertheless, there is little doubt that the decision to convene the expert panel in the first place was as much a political decision as a scientific one. Had the media attention on CCSVI been less intense the concomitant political pressure would not have materialized, and the CIHR would not have felt compelled to make a public demonstration that it was taking the issue seriously. The on-going media and political pressure was evident as well in the manner in which the panel conducted its activities. For example, several of the studies being monitored by the panel took extra-ordinary steps to send their ultra-sound technicians for special training in the Zamboni scanning protocol for venous anatomy. The concern was to avoid further controversy should these studies fail to confirm the CCSVI hypothesis, on the grounds that their imaging technique was somehow flawed such that they failed to see the confirming evidence. Again there is a certain irony when an initial study that is almost universally dismissed as methodologically flawed is used to set the standard for future studies.

Despite the panel’s best efforts to remain politically sensitive while maintaining scientific rigor, the unrelenting media and political pressure were taking a toll. It is my considered opinion that these pressures had an increasing influence on the activities of the panel. When the panel met on June 28, 2011 to review updates from the seven studies as well as the results of a meta-analysis of other CCSVI studies, it was aware that the federal minister of health was waiting in the wings to receive an update on the panel’s progress. Given that none of the seven on-going studies had definitive results to report at the time (most had not yet completed enrollment), any ostensible “progress” would be based on the meta-analysis. Those results were equivocal at best, and failed to produce “clear and convincing evidence of CCSVI,” the putative standard set at the initial scientific meeting in August 2010 [35]. Results from autopsies conducted on seven MS patients that indicated abnormal venous anatomy for some patients were also presented, although it was emphasized that these results were very preliminary, and it was too early to say what if anything this might say with regard to the CCSVI hypothesis [36]. Even when combined with the results of the meta-analysis, however, the evidence in support of the CCSVI hypothesis remained less than clear.

Any proposed clinical trial on CCSVI would most likely include a placebo arm in which participants received sham surgery. Such a trial would present its own ethical challenges with regard to the available evidentiary standard and the need to establish clinical equipoise. Even if equipoise could be established there would be issues of therapeutic misconception when patients enrolled in such a study are convinced they would receive treatment, and the resulting problems with achieving a fully informed consent [37].

I am not at liberty to go into specific details of the often animated discussion amongst panel members with regard to what to make of the evidence that had been presented. Suffice it to say that the federal Minister of Health reported the next day that the panel had endorsed a decision to proceed with a Phase I/II clinical trial [38]. Since that time CIHR has announced funding of a successful application to conduct this study [39].

In my view the pending meeting with the Minister of Health had created something of a sense of urgency about the panel’s deliberations, and an expectation that some kind of a positive announcement would be helpful. Thus the Phase I/II recommendation was in some sense both a scientific and a political compromise. Such early phase trials are generally designed to gather additional evidence in support of a promising hypothesis, and to see whether clinical equipoise can be established so as to justify a larger Phase III study. In this case, while the available evidence was equivocal at best, approving a Phase I/II study provided a means by which to forestall some of the continuing political pressure. However, this was not the full-fledged Phase III study for which advocates had been clamoring, and CIHR and the expert panel have faced continuing criticism for failing to take that step [40].

## Summary

The Canadian experience with CCSVI presents an interesting case study and raises important questions about resource allocation and priority setting in the research context. Indeed, we rarely see this kind of broad-scope public momentum behind new drugs or therapies, and we very rarely see governments so directly engaged in pushing a particular research agenda, especially at such an early stage (i.e., moving to clinical trials before there is a strong evidentiary foundation, standardized diagnostic approaches, management of the risks associated with different treatment approaches, etc. [41]).

The priority setting tensions that emerge from this reality are unquestionably complex. On what basis should we decide to fund MS research versus other high-impact diseases such as cancer or heart disease, two of the leading causes of death for Canadians? What about rare but equally devastating conditions that may not generate the same level of public advocacy, or conditions that don’t encourage the same degree of public sympathy [42]? What about other areas of MS research where the evidence is stronger? The degree to which evidence and expertise should be the determining factors in these decisions and the appropriate role for public pressure, advocacy and interest groups are all matters of considerable debate. These are by no means new challenges [43,44], but they have been brought into the spotlight again by the evolving MS liberation story [10,45,46].

One particularly salient aspect of how CCSVI has permeated political spheres is the influence of social media, not only in terms of the rapid spread of information (or misinformation), but also with its ability to mobilize large numbers of people and capture the attention of political leaders [47]. The current power of social media represents a whole new level of citizen engagement and advocacy, and emphasizes the importance of open debate about the basis on which particular resource allocation and priority setting policy choices are made in scientific and medical research contexts, especially if political leaders begin to play more direct roles in such decisions. When dealing with the allocation of limited public funds for research, decision-making should be transparent, just, and at the very least, informed by current evidence. Deliberative democracy cannot afford to be high-jacked by a cyber-mob. However, the rapidity with which new findings, whether speculative or proven, make their way into the public sphere has undergone a paradigm shift such that the process in which “evidence” is manufactured may be changing irrevocably.

Gone are the days when researchers and clinicians can rely on a few hours of “media training” to prepare them for the off-chance they might be interviewed about some aspect of their work. As the Canadian experience with CCSVI illustrates so poignantly, the advent of the internet and social media mean the ivory tower of academia might be stormed at any moment by an interested, enthusiastic, and motivated public. Researchers and clinicians must learn how to utilize these resources to ensure the message that emerges is both balanced and informed.

## Abbreviations

CCSVI: Chronic cerebrospinal venous insufficiency; CIHR: Canadian institutes of health research; CTV: Canadian television; MP: Member of parliament; MS: Multiple sclerosis; SHRF: Saskatchewan health research foundation.

## Competing interests

As noted, DP is a member of an expert panel on the topic convened by the MS Society of Canada and the CIHR; AP is a health journalist for the *Globe and Mail*, Canada’s national newspaper, and has published extensively on this topic.

## Authors’ contributions

DP conceived the concept for the paper and together with AZ developed the structure and overarching themes. Each author bears primary responsibility for his/her section – Politics and Publicity (AP); Politics and Finances (AZ); Politics and Science Policy (DP). All authors read and approved the final manuscript.

## Pre-publication history

The pre-publication history for this paper can be accessed here:

http://www.biomedcentral.com/1472-6939/14/6/prepub
